# Serological Findings in a Child with Paroxysmal Cold Haemoglobinuria

**DOI:** 10.1155/2014/316010

**Published:** 2014-10-09

**Authors:** Eduardo J. Salido, Valentín Cabañas, Mercedes Berenguer, María I. Macizo, Faustino García-Candel, Raúl Pérez-López, Jose M. Moraleda

**Affiliations:** ^1^Hematology Department, Hematopoietic Transplant and Cellular Therapy Unit, Virgen de la Arrixaca Clinical University Hospital, IMIB-Arrixaca, University of Murcia, Carretera Madrid-Cartagena, s/n, El Palmar, 30120 Murcia, Spain; ^2^Laboratory Medicine Department, Virgen de la Arrixaca Clinical University Hospital, IMIB-Arrixaca, University of Murcia, Carretera Madrid-Cartagena, s/n, El Palmar, 30120 Murcia, Spain

## Abstract

PCH is a rare autoimmune hemolytic anemia (AIHA) but is one of the most common causes of AIAH in children. For the diagnosis, it is important to perform the appropriate methods of serological investigation and show the typical biphasic reaction. This is a case report of a child who presented with features of haemolysis and was diagnosed with PCH of this way.

## 1. Introduction

Paroxysmal cold haemoglobinuria (PCH) is a special type of hemolytic syndrome caused by cold agglutinins. The hemolysin involved was described in 1904 by Donath-Landsteiner and is called Donath-Landsteiner (DL) antibody. Characteristically it is a biphasic hemolysin, and it is fixed to erythrocytes at cold temperatures and produces lysis at body temperature (37°C) [1, 2]. Complement is required for lysis but plays little or no part in the fixation phase.

PCH is one of the most common causes of acute autoimmune haemolytic anaemia (AIHA) in young children, although it may affect patients of all ages. In children, PCH is commonly seen following a viral illness or after vaccination [1, 2].

We report a child who presented with features of haemolysis and was diagnosed as PCH. We emphasize the relevance of considering PCH in presence of autoimmune hemolytic anemia with intravascular haemolysis at 37°C and a direct antiglobulin (Coombs) test (DAT) positive for complement only and implement the appropriate methods of serological investigation.

## 2. Case Report

A 3-year-old previously healthy girl was admitted to the pediatric unit with a 5-day history of viral illness with asthenia, fever, cough, and rhinorrhea. Physical examination revealed jaundice and mild hepatomegaly.

**Figure 1:**
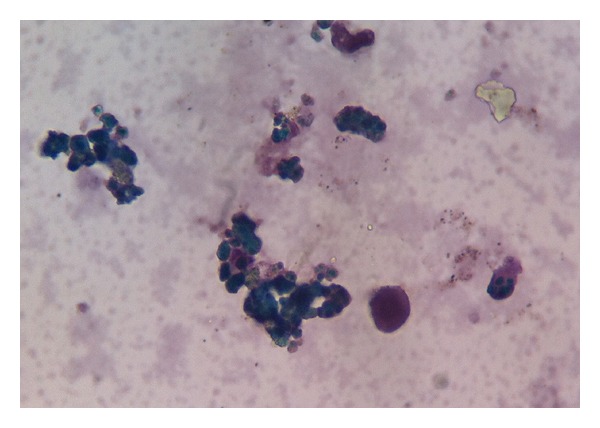
Hemosiderinuria. Perls' reaction weakly positive in the urine.

The initial investigations revealed a hemoglobin level of 75 g/L, with normal white cell and platelet count. The blood smear showed a severe red cell agglutination with moderate number of spherocytes. The reticulocyte count was initially normal. Serum indirect bilirubin was 120 *μ*mol/L with the indirect fraction of 111 *μ*mol/L and LDH was 1380 U/L. Analysis of the urine sample revealed the presence of coluria and weakly positive haemosiderin (Perls' reaction, Figure 1) indicating intravascular haemolysis, with absent bilirubin, and no red cells were seen under microscope. Renal function was normal. These results pointed towards an intravascular haemolysis.

We found a positive direct antiglobulin test (DAT) due to primarily complement sensitization (+4) with C3d specificity and IgG was negative.

The cold agglutinin titre was not significant with adult cells, cord cells, and patient's cells. However, the Donath-Landsteiner test was positive which was directed towards the diagnosis of PCH.

In view of finding of an underlying cause, serological investigations were performed to discard infections (mycoplasma and EBV were negative) and serological investigations to detect Donath-Landsteiner hemolysin.

## 3. Serological Investigations in PCH 

The diagnosis of PCH requires the demonstration of the typical serological reaction of Donath-Landsteiner hemolysin.

The DL antibody of PCH is an IgG antibody and has specificity for P antigens. It is far more lytic to normal cells in relation to its titre than the anti-I or anti-i antibodies. The lysis titre of a DL antibody may be the same as or greater than its agglutination titre. The maximal lysis develops in unacidified serum and this is inhibited by plasma [2].

### 3.1. Direct Donath-Landsteiner Test

We collected two samples of venous blood into tubes without anticoagulant, previously warmed at 37°C. Later we incubated the first sample at 37°C for 1,5 hours. The second sample was incubated in a container filled with ice for 1 hour and afterwards was placed at 37°C for another 20 minutes. After the incubation time, both samples were centrifuged at 37°C and the supernatant serum was examined for lysis.

**Figure 2:**
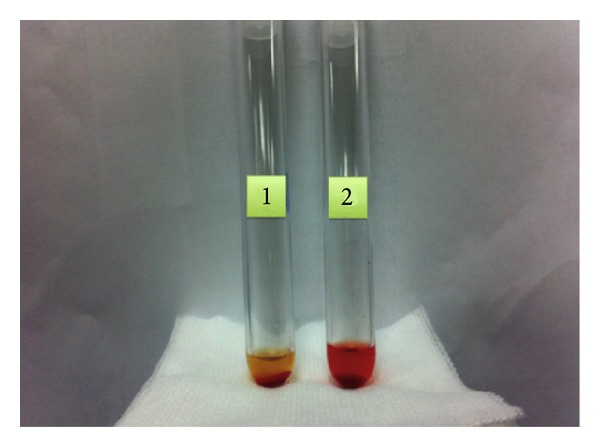
Direct Donath-Landsteiner positive test.

The test was positive for the presence of hemolysis in the sample that had been chilled (Figure 2, tube 2). Although the test was positive, we proceeded to confirm the presence of antibody by indirect Donath-Landsteiner test.

### 3.2. Indirect Donath-Landsteiner Test

**Figure 3:**
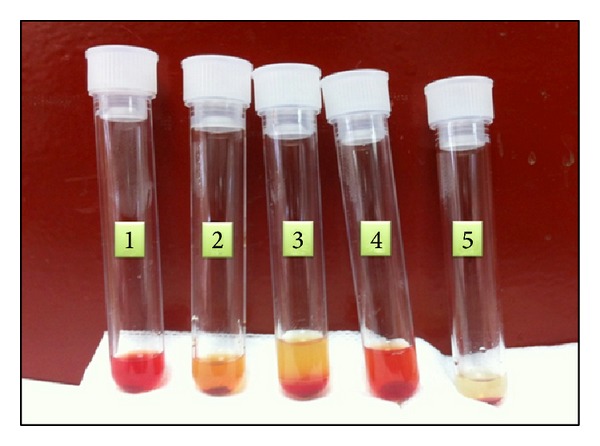
Indirect Donath-Landsteiner test. Tube 1: OP + red cells suspension + patient's serum. Double incubation at 0°C and 37°C. Tube 2: duplicate of tube 1 but conserved strictly at 37°C. Tube 3: duplicate of tube 1 + compatible fresh normal EDTA-plasma and double incubation. Tube 4: duplicate of tube 1 + compatible fresh normal serum and double incubation. Tube 5: OP + red cells suspension + ABO compatible fresh serum and double incubation.

We performed the indirect DL tests using serum obtained from the patient's blood that had been allowed to clot at 37°C. We added 1 volume of 50% suspension of washed normal group O, P-positive red cells to 9 volumes of the patient's unacidified serum in a tube. Then we chilled the suspension in crushed ice at 0°C for 1 hour. Afterwards we placed the tube at 37°C for 30 minutes, centrifuged it at 37°C, and examined it for lysis (Figure 3, tube 1).

At the same time we used three control tubes:

- a duplicate tube of the test cell-serum suspension (tube 1) but conserved strictly at 37°C for the duration of the test (Figure 3, tube 2);
- the adsorption of complement that can be prevented by adding a chelating agent, such as EDTA, to the serum. In this case there is no hemolysis, showing that reaction is complement dependent (Figure 3, tube 3);
- a duplicate of the test cell-serum suspension, except that an equal volume of ABO-compatible fresh normal serum was first added to the patient's serum as a source of complement. One volume of the 50% cell suspension was added and the suspension chilled and subsequently warmed in the same way as the test suspension. This control excludes false negative results owing to the patient's serum being deficient in complement (Figure 3, tube 4);
- a duplicate of the test cell-serum suspension, except that fresh normal serum was used in place of the patient's serum. This control also was chilled and subsequently warmed.

## 4. Discussion

PCH was first described as a distinct clinical entity in 1872 and the hemolytic antibody was described by Donath and Landsteiner. PCH is an intravascular hemolytic disease in which hemolysis is related to exposure to cold temperatures [1]. The DL hemolysin is an IgG cold antibody and has anti-P specificity. However, in practice, almost all samples of red cells are suitable because the cells that will not react (Pk and pp) are extremely rare [2, 3].

The incidence of PCH according to the data available is variable from 5 to 10% of autoimmune hemolytic anemia (AIHA) in children [1] and is one of the most common causes of acute AIHA in the young children [1]. In adults, PCH is rare, representing less than 1% of all AIHA [1]. A study found six cases of PCH in 100 children with AIHA [4].

The antibody is fixed on the erythrocytes at low temperature, and hemolysis later occurs at body temperature. Complement is necessary for hemolysis but not for fixation [2, 3].

The antibody was first observed as a cross-reacting antibody to an antigen on* Treponema pallidum *in the latter half of the 19th century. Today it is more common to find it in children with postviral illnesses [5], after immunization [6], and in patients with lymphoproliferative disorders and collagen vascular diseases [7].

Attacks of hemoglobinuria are precipitated by cold and hemolysis may occur immediately after exposure and is usually accompanied by fever, abdominal pain, and constitutional symptoms that resolves spontaneously within a few days to several weeks after onset [1, 5].

Regarding the laboratory findings, it is striking the relative reticulocytopenia in the presence of severe and rapidly progressive anaemia. Reticulocytopenia reflects an ineffective erythropoiesis either by destruction of precursor red cells by Donath-Landsteiner antibodies or by viral hemopoietic suppression [1, 4].

During the hemolytic crisis there may be methemoglobinemia and hemoglobinuria, the concentration of haptoglobin is reduced, and the LDH levels are greatly increased [1, 4].

Direct antiglobulin tests are usually positive during a paroxysm and negative at other times and it is complement and temperature dependent [1, 4].

Leukopenia occurs during or immediately after a paroxysm, followed by leukocytosis. In the blood smear it is common to observe spherocytosis, anisopoikilocytosis, fragmented red cells, basophilic stippling, polychromatophilia, autoagglutination, and nucleated erythrocytes [1, 4].

In our patient, urine haemosiderin positivity confirmed intravascular haemolysis and the C3d specificity supported an immune-mediated haemolysis mechanism.

To establish the correct diagnosis of PCH, it is necessary to demonstrate the presence of the DL hemolysin using appropriate serological investigations as we have described, using both negative and positive controls. The Donath-Landsteiner reaction is positive at all stages of the disease and may even persist for years, although during remission the hemolytic activity usually may be minimal [2].

In our patient, the cold agglutinin titre was also performed and it was not significant.

Determination of the specificity of the autoantibody is recommended, but it was not performed in our patient because almost all cases of PCH are associated with anti-P specificity, although other specificities associated with PCH have been rarely reported [1]. In practice it is impossible to determine the specificity of antibody due the great rarity of P negative cells [6].

Though acute attacks are normally severe, they characteristically resolve spontaneously within a few days or few weeks after the onset and rarely recur [1]. Our patient required only supportive care that included blood transfusion and warming.

The suspected diagnosis is important because corticosteroids are usually ineffective and unnecessary because of the transient nature of haemolysis [1]. Some adults have been treated with cyclophosphamide, rituximab was used successfully in rare refractory cases, and clearance of the antibody may be possible with plasma exchange [7, 8].

In conclusion, in patients with suspected PCH it is necessary to perform a correct diagnosis by serological demonstration of DL hemolysin because management may be different from other hemolytic anemia.
